# A spatially-explicit database of tree-related microhabitats in Europe and beyond

**DOI:** 10.3897/BDJ.10.e91385

**Published:** 2022-10-12

**Authors:** Sergey Zudin, Wilfried Heintz, Daniel Kraus, Frank Krumm, Laurent Larrieu, Andreas Schuck

**Affiliations:** 1 European Forest Institute, Joensuu, Finland European Forest Institute Joensuu Finland; 2 INRAE, UMR Dynafor, Castanet-Tolosan, France INRAE, UMR Dynafor Castanet-Tolosan France; 3 INP Toulouse, ENSAT, EI Purpan, Toulouse, France INP Toulouse, ENSAT, EI Purpan Toulouse France; 4 Bavarian State Forest, Neureichenau, Germany Bavarian State Forest Neureichenau Germany; 5 Swiss Federal Institute for Forest, Snow and Landscape Research, Brimensdorf, Switzerland Swiss Federal Institute for Forest, Snow and Landscape Research Brimensdorf Switzerland; 6 Université de Toulouse, INRAE, UMR Dynafor, Castanet-Tolosan, France Université de Toulouse, INRAE, UMR Dynafor Castanet-Tolosan France; 7 5CNPF-CRPF Occitanie, Toulouse, France 5CNPF-CRPF Occitanie Toulouse France

**Keywords:** TreMs, tree species, Europe, spatially explicit, biodiversity

## Abstract

**Background:**

Tree to tree interactions are important structuring mechanisms for forest community dynamics. Forest management takes advantage of competition effects on tree growth by removing or retaining trees to achieve management goals. Both competition and silviculture have, thus, a strong effect on density and distribution of tree related microhabitats which are key features for forest taxa at the stand scale. In particular, spatially-explicit data to understand patterns and mechanisms of tree-related microhabitats formation in forest stands are rare. To train and eventually improve decision-making capacities related to the integration of biodiversity aspects into forest management plot of one hectare, so called marteloscopes were established in the frame of the ‘European Integrate Network’. In each plot, a set of data is collected at the individual tree level and stored in a database, the ‘I+ repository’. The 'I+ repository' is a centralised online database which serves for maintaining the data of all marteloscope plots. A subset of this repository was made publicly available via the Global Biodiversity Information Facility, based on a data-sharing policy. Data included are tree location in plot, tree species, forest mensuration data (diameter at breast height [cm], tree height [m]), tree status (living or standing dead) and tree-related microhabitats. Further, a visual assessment of timber quality classes is performed in order to provide an estimate of the economic value (market price) for each tree. This information is not part of the GBIF dataset.

**New information:**

Currently 42,078 individual tree observations from 111 plots are made available via the Global Biodiversity Information Facility (GBIF). As the network of plots continues to expand, so does the database of tree-related microhabitats. Therefore, the database will undergo a regular update. The current version has a temporal coverage from March 2014 to December 2020. The innovation of this unique dataset is that it is based on a commonly agreed catalogue of tree microhabitats as a field reference list when assessing assessment protocol. The reference list is available in 17 languages and, thus, helps to guarantee compatibility of tree-related microhabitat assessments across countries and plots.

## Introduction

Tree-related microhabitats (hereafter called TreMs) are ecological objects defined as "distinct, well-delineated structures occurring on living or standing dead trees, that constitute particular and essential substrates or life site for species or species communities during at least a part of their life cycle to develop, feed, shelter or breed" (Larrieu et al. 2018). These authors narrowed the TreM definition to focus on morphological singularities located above-ground, excluding singularities borne in lying deadwood items, as well as generic tree species-specific characteristics.

### TreMs as pivotal ephemeral resource patches for a wide range of taxa

TreMs can be considered as "ephemeral resource patches", i.e. spatially and temporally delimited patches of high quality resource (Finn 2001). They are usually small in size and also limited in their extent by the dimensions of the bearing-tree. Even though certain TreMs are relatively long-lasting (e.g. large rot-holes) and can last decades, TreMs are temporary structures: a TreM can either disappear if the bearing-tree is removed, it evolves to another type given there are different development conditions or if the tree dies. A TreM can also be periodically unavailable, such as water-filled holes which are used by associated species only when filled with water. TreMs provide a wide range of specific conditions including variations in microclimates and substrates. Furthermore, certain TreMs can supply different conditions depending on the period of the year. TreMs serve many purposes: they can be shelter, foraging or reproduction sites and, for some species, provide all vital functions for their full life cycle. Base rot-holes on an oak, for example, can supply a habitat for the full life cycle of beetles (Gouix 2011) and be used as a simple temporary shelter by rodents (Le Louarn and Quéré 2003). Therefore, there exists a dependence gradient of species to TreMs. TreMs are used by a large variety of taxa, from animals to vascular plants, bryophytes, fungi and lichens (Larrieu et al. 2018).

### TreMs participate in a complex habitat functional network

Many species called "multi-habitat species" (Van Halder et al. 2008) require different resources to meet all of their vital needs. These so-called "complementation resources" (Tilman 1982) affect both the size of the population and its persistence (Dunning et al. 1992), as well as the spatial distribution of individuals at a stage of development, conditioned to the requirements of individuals at another stage (Ockinger 2008). Two modalities of such resources often concern TreMs. Firstly, the availability of two eco-phases for a particular species for example, flowers for adults and mould inside rot-holes for larvae of hoverflies (Speight et al. 2020). Secondly, they can be a resource required by the same eco-phase, for example, water bodies for cavity-dwelling bats that need to drink before hunting (Arthur and Lemaire 2009). Additionally, species may use several TreMs of the same type or which provide the same function, available in its range of action. Such “supplementation resources” (Tilman 1982) will improve the availability of required habitats and, thus, contribute to maintaining, or even increasing, population densities of particular species (Dunning et al. 1992). Spatial distribution of these complementation and supplementation resources is essential for species which depend on them. In order to provide full potential, resources need to be connected, i.e. closer than the dispersal or prospecting range of the individuals and separated from the primary resource by a permeable matrix (Dunning et al. 1992). This is important as many TreM-dwelling species have rather low dispersion capacities (Ranius and Hedin 2001). TreMs also play a pivotal role in increasing the ecological complexity of a forest habitat. Ecological complexity favours high specific richness (Rosenzweig 1995), which is essential for the stability of ecosystem services in changing environments (Loreau et al. 2001), especially as species may respond differently to environmental variations (Yachi and Loreau 1999). A large structural heterogeneity of forest stands will also increase the number of functional groups (Huston 1994).

### TreMs are keystone structures for forest ecosystems

**Figure 1.**
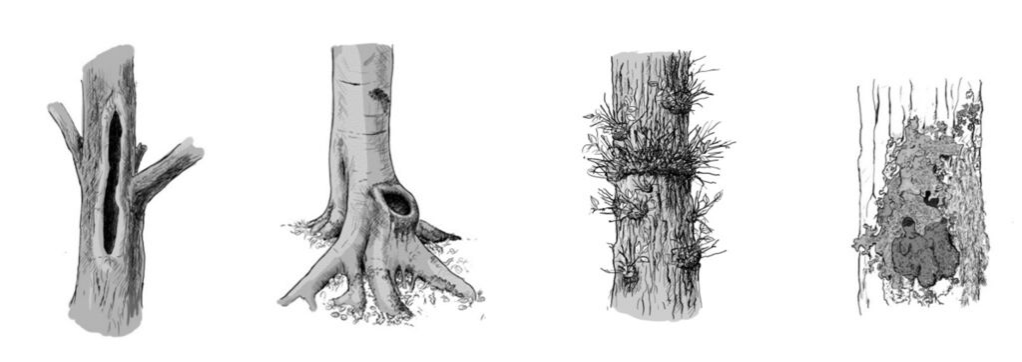
Selected tree-related microhabitat structures. From left to right: rot-hole, dendrotelm, epicormic shoots, epiphytic foliose and fruticose lichens (Kraus et al. 2018).

TreMs provide multiple ecological habitat functions for a large number of species that are associated with them. Therefore, they play a pivotal role in conserving species diversity in forest ecosystems. Facilitating functional redundancy (Huston 1994), a high level of biodiversity likely contributes to increasing productivity, resistance and long-term resilience of forest ecosystems (Thompson et al. 2009). Providing resources, shelter or goods and services crucial for particular species groups throughout a distinct spatial structure, TreMs can be considered as “keystone structures” (Tews et al. 2004) for forest ecosystems (Fig. 1).

### TreMs are biodiversity indicators for conservation issues

Several authors suggested using TreMs as biodiversity indicators in forest ecosystems and as tools to promote biodiversity within managed forests (Winter and Möller 2008, Bütler et al. 2013, Regnery et al. 2013, Larrieu et al. 2018, Paillet et al. 2018) although further research is required to better quantify relationships between TreMs and taxa at the stand scale (Asbeck et al. 2021).

### Why is a database on TreMs crucial for research?

Borne by only a fraction of trees within forest stands, most of TreMs are, therefore, rare events. Still, actual TreM occurrence can differ, for example, due to stand development or age, thus being more common in unmanaged old-growth forests with high structural complexity as compared to young managed forest stands. In order to perform statistically sound analyses, the need for a large and standardised dataset is evident. Therefore, large standardised datasets are needed for performing statistically-sound analyses. Having available extensive number of trees individually observed not only across a wide range of forest types and biogeographical regions, but also a variety of management intensities (from old-growth forests to recently-harvested stands), makes this database a significant contribution to this field of research. As all trees are georeferenced, also the spatial distribution of TreMs can be investigated, providing new insights for understanding relationships between TreMs and TreM-dwelling taxa. This database has been used, for example, to investigate the co-occurrence patterns of TreMs (Larrieu et al. 2021) and modelling the rate of TreM formation on living trees (Courbaud et al. 2021).

## Geographic coverage

### Description

The network of marteloscope plots subject to this database is almost exclusively located in Europe. It is, however, open to include plots from institutions around the world recording data based on the collection protocol for tree-related microhabitats. So far, plots are included from the following European countries: Belgium, Bosnia and Herzegovina, Czech Republic, Denmark, France, Germany, Hungary, Ireland, Italy, Luxembourg, Poland, Serbia, Slovakia, Slovenia, Spain, Sweden and Switzerland. A few datasets are also from other world regions, namely Chile and Iran.

### Coordinates

-41.64 and 69.3 Latitude; -73.92 and 57.31 Longitude.

## Taxonomic coverage

### Description

Included in the spatially-explicit database of tree-related microhabitats are 89 species (Table 1). The number of observations by species varies from 1 (*Cornus*, *Juglans*, *Ostrya*, *Pawlonia*) to 14791 (*Fagus* spp.).

## Usage licence

### Usage licence

Other

### IP rights notes

Creative Commons Attribution (CC-BY)

## Data resources

### Data package title

Spatially-explicit database of tree-related microhabitats (TreMs)

### Resource link

https://www.gbif.org/dataset/2e102194-f384-4712-89a4-5db7a3fc409a

### Number of data sets

1

### Data set 1.

#### Data set name

Spatially-explicit database of tree-related microhabitats (TreMs)

#### Data format

Darwin Core Archive (DwC-A)

#### Download URL

http://dynids.toulouse.inra.fr:8180/ipt/archive.do?r=trems_integrate

#### Description

The ‘Spatially-explicit database of tree-related microhabitats (TreMs)’ is derived from the ‘I+ repository’. It includes all trees above the defined minimum diameter of 7.5 cm at breast height (1.30 m), both exhibiting or lacking TreMs. The dataset structure is based on Darwin Core Standard (maintained by TDWG), which provides a stable standard reference for sharing information on biological diversity. There are two files in DWC-A: occurrence.txt (trees data) and measurementorfact.txt (trems data). Both tab delimited. Total number of columns equal 30.

**Data set 1.** 

Column label	Column description
ID	GBIF tree ID.
language	Dataset language (’en’).
accessRights	Access rights (’open access’).
datasetID	Dataset ID (doi): https://doi.org/10.15468/ocof3v
datasetName	Dataset name (‘trems dataset’).
basisOfRecord	Type of recording (’ HumanObservation’).
occurrenceID	I+ tree ID (treeId_Iplus_2AlfaCountryCode-PlotName).
eventDate	Year of observation.
habitat	Type of forest community. Example : ‘Beech-oak'.
country	Country name.
verbatimElevation	Elevation.
verbatimCoordinates	tree cordinates in plot.
verbatimCoordinateSystem	Marteloscope’s coordinate system (‘decimal degrees').
decimalLatitude	Marteloscope’s latitude.
decimalLongitude	Marteloscope’s longitude.
geodeticDatum	DATUM (WGS84).
coordinateUncertaintyInMetres	Coordinates uncertainty in metres.
identificationID	Unique record id.
identificationRemarks	Identification remark: 'uncertain' if scientific name equal 'PLANTAE' (tree species unknown).
scientificName	Tree species. Tree species are provided by their scientific name. Note that dead standing trees are also recorded with tree species designation.
genus	Genus.
specificEpithet	Species part of scientific name.
taxonRank	Lowest determined taxon rank (species/genus/kingdom).
id (measurementorfact.txt)	Occurrence id - equal to OccurrenceID (treeId_Iplus_2AlfaCountryCode-PlotName).
measurementType	Trems code : based on the ‘Catalogue of Tree Microhabitats - Field Reference List’ (Kraus et al. 2016). The catalogue comprises 64 saproxylic (encompassing decaying wood) and epixylic (without decaying wood) microhabitat types, such as cavities, large dead branches, cracks and loose bark, epiphytes, sap runs or trunk rot characteristics. The TreM types are specified by unique alphanumerical codes, for example, CV22 being ‘trunk and mould cavities ø ≥ 30 cm (ground contact); in case of other tree variables, these can be tree height, tree diameter.
measurementValue	Abundance, or physical value for tree height or diameter.
measurementAccuracy	Accuracy (for tree height and tree diameter only).
measurementUnit	Units of measurement: abundance in case of TreMs or physical unit (cm, m) for DBH and height.
measurementMethod	Measurement method reference: for TreMs reference to Catalogue, obtained height and diameter - instruments used.
measurementRemarks	For TreMs - Catalogue code, for others - name of measured variable.

## Additional information

**Figure 2.**
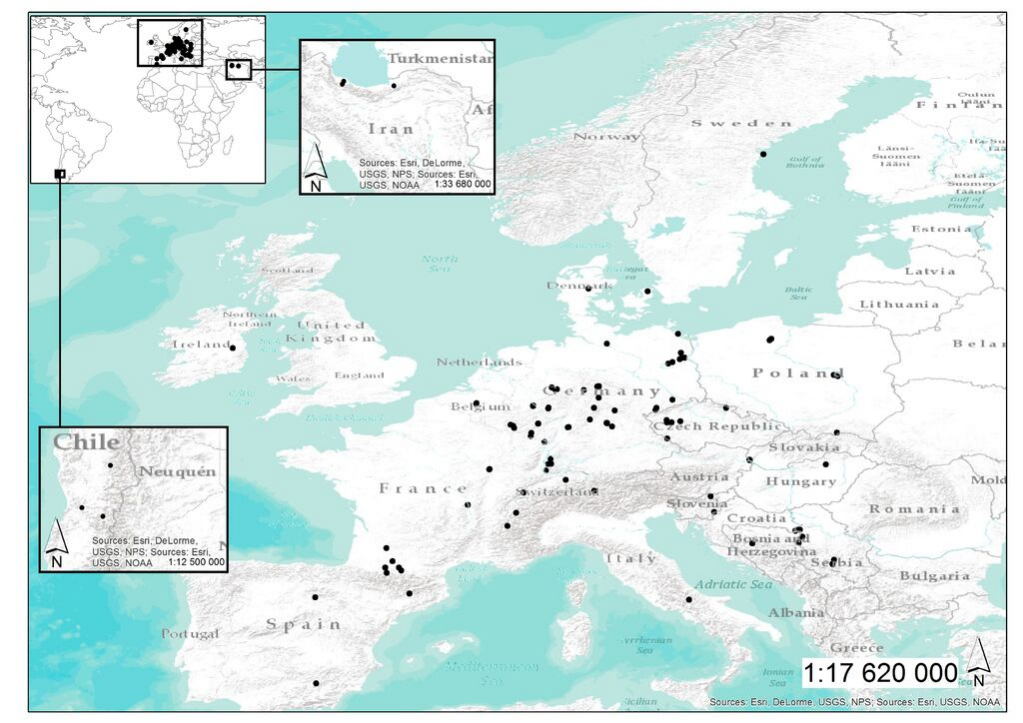
Geographic distribution of plots available in the ‘Spatially-explicit database of tree-related microhabitats (TreMs)’.

**Figure 3.**
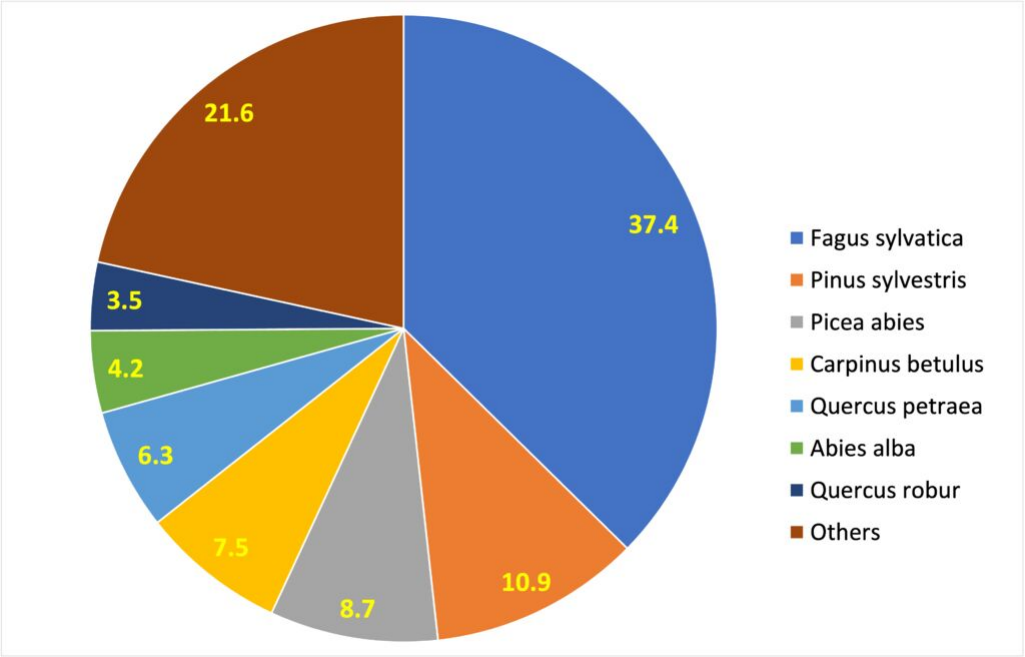
Share of main tree species in the TreMs database.

**Figure 4.**
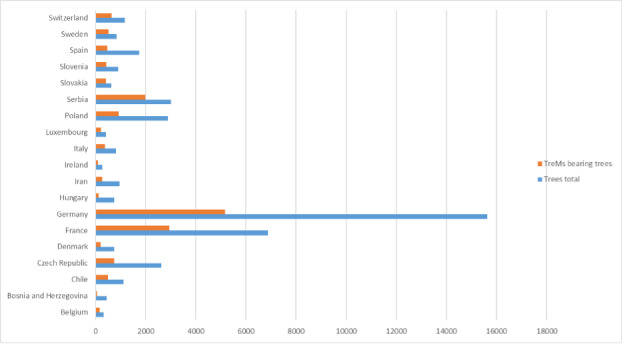
Total number or recorded trees as compared to those bearing tree-related microhabitats by country.

**Figure 5.**
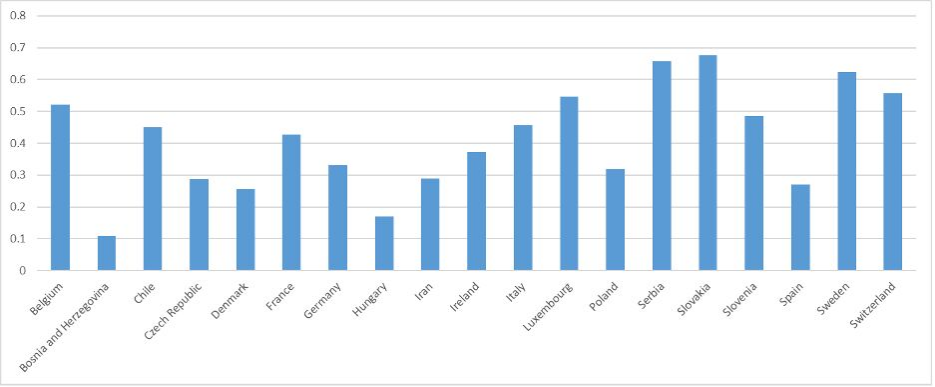
Ratio between all recorded trees and trees bearing at minimum one tree-related microhabitat by country.

**Figure 6.**
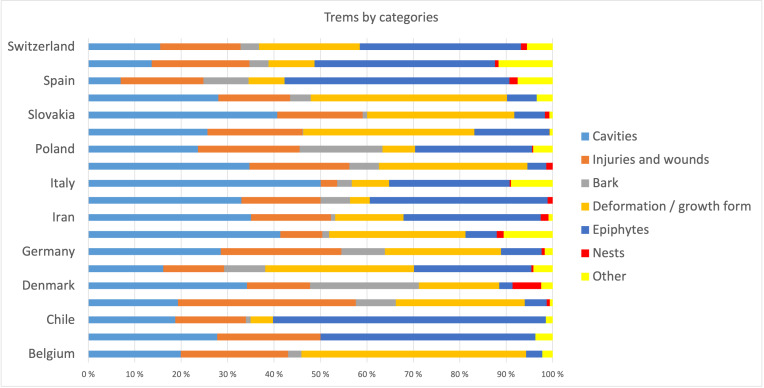
Distribution of tree-related microhabitats by main categories and countries.

**Figure 7.**
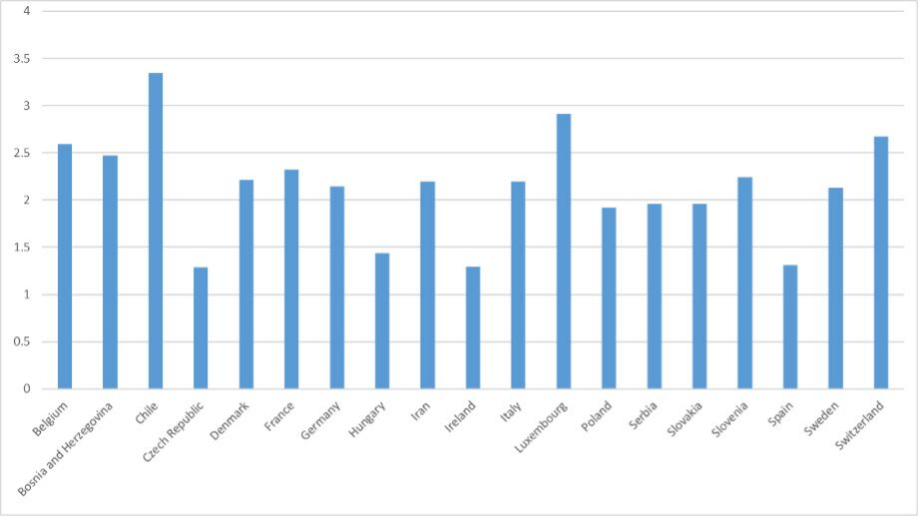
Average number of tree-related microhabitats by individual bearing tree and country.

The ‘spatially-explicit database of tree-related microhabitats (TreMs)’ comprises data of 111 plots distributed across in 19 countries and total number 42,078 trees (Fig. 2) (Kraus et al. 2021). The individual plots are mainly located in public and community forests, but have been established also in church forests and privately-owned forests. They were selected by the forest owners, based on representing a particular forest management type. The number of plots differs widely between countries (Table 2). Each individual plot is described in more detail in an information sheet which can be accessed at: http://iplus.efi.int/. The plots in Bosnia-Herzegovina (1), Chile (3) and Iran (3) were set up to monitor TreMs occurrences only and have no further site description. Data collection in all plots followed the agreed assessment protocol for TreMs as published in (Kraus et al. 2016). TreMs surveys were conducted from the ground using binoculars, assuring good light conditions. TreMs recording in broadleaved forest stands were implemented without foliage during the winter months. Fig. 3 provides insight into the share of the most commonly recorded trees species by genus in the TreMs database. Most common are *Fagussylvatica* (37.3%), *Pinussylvestris* (10.9%), *Piceaabies* (8.7%), *Carpinusbetulus* (7.5%) and *Quercuspetraea* (6.3%). When looking at trees bearing at least one TreM, we find 16,233 entities. As an individual tree may host more than one TreM, the total number of recorded TreMs amounts to 34,228. Fig. 4 gives an overview on the total number of recorded trees by countries as compared to those bearing TreMs, while Fig. 5 shows the ratio of trees by country bearing at minimum one TreM. The number of TreMs recorded on a plot may vary considerably due to, for example, the given tree species composition, stand structure, stand age or management regimes (including long-time unmanaged forests). Thus, there are variations from 0.1 to nearly 0.7, while the overall average across all countries and plots is about 0.4. Fig. 6 presents the distribution of TreMs by main categories. Each of the main categories is further divided into subcategories as described in (Kraus et al. 2016). The average number of TreMs by individual host tree varies from nearly 1.3 in the Spanish to nearly 3.4 in Chilean plots (Fig. 7). For all plots in the database, two TreMs are found on average for each TreM-bearing tree.

## Figures and Tables

**Table 1.** Listing of all tree species occurring in the spatially-explicit database of tree-related microhabitats.

**Rank**	**Scientific name**	**Rank**	**Scientific name**
species	*Abiesalba*	species	*Parrotiapersica*
species	*Abiesgrandis*	species	*Paulowniatomentosa*
species	*Acercampestre*	species	*Persealingue*
species	*Acercappadocicum*	species	*Piceaabies*
species	*Acerlobelii*	species	*Piceasitchensis*
species	*Aceropalus*	species	*Pinuscembra*
species	*Acerplatanoides*	species	*Pinusmugo*
species	*Acerpseudoplatanus*	species	*Pinusnigra*
species	*Acertataricum*	species	*Pinuspinaster*
species	*Acervelutinum*	species	*Pinusstrobus*
species	*Aesculushippocastanum*	species	*Pinussylvatica*
species	*Aextoxiconpunctatum*	species	*Podocarpusnubigena*
species	*Alnusglutinosa*	species	*Populustremula*
species	*Alnusincana*	species	*Prunusavium*
species	*Alnussubcordata*	species	*Prunuspadus*
species	*Amomyrtusluma*	species	*Prunusserotina*
species	*Araucariaaraucana*	species	*Prunusspinosa*
species	*Betulapendula*	species	*Pseudotsugamenziesii*
species	*Betulapubescens*	species	*Quercuscerris*
species	*Caldcluviapaniculata*	species	*Quercusfaginea*
species	*Carpinusbetulus*	species	*Quercusfrainetto*
species	*Castaneasativa*	species	*Quercusilex*
species	*Cornusmas*	species	*Quercuspetraea*
species	*Corylusavellana*	species	*Quercuspubescens*
species	*Corylusmaxima*	species	*Quercusrobur*
species	*Crateagusmonogyna*	species	*Quercusrubra*
species	*Diospyroslotus*	species	*Robiniapseudoacacia*
species	*Eucryphiacordifolia*	species	*Salixcaprea*
species	*Fagusorientalis*	species	*Sambucusnigra*
species	*Fagussylvatica*	species	*Sorbusaria*
species	*Frangulaalnus*	species	*Sorbusaucuparia*
species	*Fraxinusexcelsior*	species	*Sorbusdomestica*
species	*Fraxinusornus*	species	*Sorbustorminalis*
species	*Gevuinaavellana*	species	*Taxusbaccata*
species	*Ilexaquifolium*	species	*Tiliabegonifolia*
species	*Juglansregia*	species	*Tiliacordata*
species	*Juniperusoxycedrus*	species	*Tiliaplatyphylla*
species	*Larixdecidua*	species	*Tiliatomentosa*
species	*Larixkaempferi*	species	*Tsugaheterophylla*
species	*Laureliasempervirens*	species	*Ulmuscanescens*
species	*Laureliopsisphilippiana*	species	*Ulmusglabra*
species	*Malussylvestris*	species	*Ulmuslaevis*
species	*Nothofagusalpina*	species	*Ulmusminor*
species	*Nothofagusdombeyi*	species	*Weinmanniatrichosperma*
species	*Ostryacarpinifolia*		

**Table 2.** Distribution of plots by countries.

**Country**	**Number of plots**	**Country**	**Number of plots**
**Belgium**	2	**Italy**	1
**Bosnia and Herzegovina**	1	**Luxembourg**	3
**Chile**	3	**Poland**	5
**Czech Republic**	6	**Serbia**	14
**Denmark**	2	**Slovakia**	2
**France**	12	**Slovenia**	2
**Germany**	46	**Spain**	3
**Hungary**	1	**Sweden**	1
**Iran**	3	**Switzerland**	3
**Ireland**	1		
